# Within-Subject Performance on a Real-Life, Complex Task and Traditional Lab Experiments: Measures of Word Learning, Raven Matrices, Tapping, and CPR

**DOI:** 10.5334/joc.65

**Published:** 2019-05-23

**Authors:** Florian Sense, Sarah Maaß, Kevin Gluck, Hedderik van Rijn

**Affiliations:** 1 Department of Experimental Psychology, University of Groningen, NL; 2 Behavioral and Cognitive Neurosciences, University of Groningen, NL; 3 Air Force Research Laboratory, Wright-Patterson Air Force Base, OH, US

**Keywords:** Long-term memory, Executive functions, Learning, Memory, CPR

## Abstract

In this data report, we describe a three-session experiment spanning six months. Several well-controlled laboratory tasks (Word Learning, Raven Matrices, and Tapping) and Cardiopulmonary Resuscitation (CPR), a complex but well-defined real-world task, were administered. Data are reported from 50 participants for the first session, 40 for the second, and 34 for the third. CPR is a useful domain for studying real-world performance inside the laboratory because clear performance standards can be applied to quantifying learners’ proficiency covering both the first steps that need to be taken prior to the initiation of CPR (declarative knowledge) as well as the compressions and ventilations themselves (procedural skill). This research resulted in a rich dataset with a range of different measures for all participants. For all tasks, the complete set of raw data are made available along with relevant aggregate performance scores (see https://osf.io/m8bxe/). The raw data in particular will enable other researchers to explore potential analyses and modeling beyond the scope of our own. The details of the data collection protocol and available data are documented here to facilitate this process.

## Introduction

Psychologists routinely study learning, forgetting, problem solving, and other cognitive tasks of various complexities in the lab. Usually, laboratory tasks are designed to create a tightly controlled environment to scrutinize a single aspect of the process under investigation. We had the opportunity to collect data that combined three such well-controlled, single-purpose tasks (Word Learning, Raven Matrices, and Tapping) along with a complex, real-world task: Cardiopulmonary Resuscitation (CPR). CPR is a useful domain for studying a real-world task inside the laboratory because behavior is highly scripted, with individuals clearly differing in their performance and the speed by which it deteriorates (McKenna & Glendon, 1985), but clear performance standards that can be applied to quantifying learners’ proficiency (Perkins et al., 2015). The guidelines describe the steps that need to be taken prior to the initiation of CPR (relying on declarative knowledge), as well as rules for the frequency and force with which compressions and ventilations need to be performed (relying on procedural skills). Due to modern, instrumented CPR manikins, precise data on procedural performance are available, which allows modelling the behavioral profiles at high temporal resolution. The accuracy of the initial steps can be scored by the experimenter so the entire spectrum of CPR performance is quantified.

This research resulted in a rich dataset with a range of different measures for all participants. For all tasks, the complete set of raw data are made available along with relevant aggregate performance scores (see https://osf.io/m8bxe/). In the following, we will provide a brief overview of the administered tasks and what motivated their inclusion. The following sections in this document provide the *data collection protocol* and a complete overview of the *available data*.

We selected three lab tasks that might capture individual differences in the declarative and procedural aspects of CPR performance. The word learning task uses an adaptive learning algorithm that has been shown to be a reliable measure of retention of declarative knowledge (Sense, Behrens, Meijer, & van Rijn, 2016). In addition to the individual responses to each item – when are items presented, accuracy and latency of each response – we also report an overall rate of forgetting per participant, providing an index to their declarative knowledge skills. Earlier work suggests that the rate of forgetting is not simply a function of general cognitive ability or working memory capacity (Sense, Meijer, & van Rijn, 2018), which is why we included a dedicated proxy for general cognitive ability: a variation of the Raven matrices task (Raven, 2000). The synchronization and continuation tapping task (Semjen, Schulze, & Vorberg, 2000) was included to measure participants’ capacity to coordinate perception of and action to a rhythmic pattern. Coordinating perception and action is relevant in the context of CPR as compressions need to be administered at a specific and stable rate for them to be clinically effective. We fixed tapping rates at 110 bpm in one block because it is the mean of the recommended compression rate (100–120 bpm). Furthermore, the instructional video showcased compressions being administered to the beat of “Staying Alive” by the Bee Gees (103 beats per minute; bpm) to provide a cue that participants can “synchronize” with when initiating compressions and later need to “continue” during CPR.

In this manuscript, we document in detail how all data were collected. Our goal is to enable other researchers to exploit these data and we believe that they are especially interesting to those interested in within-subject fluctuations in performance, both within and across sessions. The sample size is relatively small, which makes computing correlations between aggregate measures problematic. However, there is a wealth of data for each participant, allowing a range of exploratory analyses. For example, one could assess the external validity of the synchronization and continuation task by linking detailed measures of moment-to-moment variability to the variability observed in the compression data, or determine motor-noise patterns at an individual level to correlate them with the confidence by which memory statistics were determined on the basis of keystroke latency data. In addition, the detailed CPR data (see Table 1) lend themselves to an investigation of which types of errors participants make when re-learning CPR and which aspects of performance suffer more than others after a delay: Are participants as likely to forget the correct compression depth as they are to forget the correct hand placement? And are these patterns mediated by intelligence and/or the rate of forgetting? Many other potential questions could be explored and we hope the details in this report facilitate this process.

**Table 1:** Information available for each recording session of CPR testing and practice. Terms in *italics* correspond to variable labels in the raw data files.

Category (*type*)	Rows	Description of category information	Example *type2* information

*CprSessionInfo*	14	Info about manikin settings and start and end times of each recording.	*totalTime*
*CprSessionStatistics*	76	Means and SDs for performance metrics and information for visuals displayed on SimPad.	*compCount, ventMeanVolume, ventTooMuchVolumePercentage*
*CprSessionScore*	23	Overall score, compression, and ventilation scores, and score reductions applied.	*overallScore, ventOverallScore, compRateHighScoreReduction*
*CprInactivityCPR*	variable	Coding of the inactivity between compression and ventilation events.	*compInactivity*
*CprCompEvent*	variable	Timestamped details on each recorded compression.	*compDepth, compReleaseDepth*
*CprVentEvent*	variable	Timestamped details on each recorded ventilation.	*ventVolume*

## Data Collection Protocol

**Figure 1:**
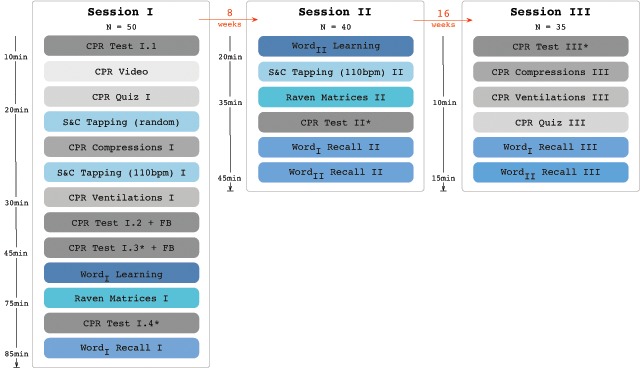
Overview of the data collection protocol. Asterisks (*) indicate that participants were trained until CPR performance was above criterion (>75%).

In the following we detail the data collection protocol and provide an overview of the data available for each administered task. Figure 1 gives a visual overview of the administered tasks and the logic of the labelling used.

### Participants

A total of 50 participants (37 female; 74%) with a median age of 20 (SD = 1.84)^1^ were recruited from the participant pool at the University of Groningen. Of these, 10 dropped out after the first session, and a further 5 after the second, leaving 35 participants with data for all three sessions.^2^

Requirement for recruitment was the possession of a valid German driver’s license (because of the requirement in Germany to complete first aid training before obtaining the license—ensuring that participants had been trained previously). Two participants indicated minimal familiarity with Swahili, which is relevant to the paired associates word learning task in this study. All participants gave written informed consent for each session and the study was approved by the Western Institutional Review Board (protocol #20151567) and the local Ethics Committee Psychology (ID: 17017-S-NE(a,b)).

### Procedure and Stimuli

Participants completed three experimental sessions with delays of 8 weeks between Sessions I and II, and 16 weeks between Sessions II and III. At the beginning of each session, each participant signed an informed consent form. A graphical summary of the paradigm is provided in Figure 1, indicating the timing both within and between sessions. Each of the components are described in more detail below.

#### Session I

##### CPR Test I.1

In the first session participants entered the experimentation room where a Laerdal Resusci Anne® QCPR manikin^3^ was lying on the ground. Participants read the following instructions: “*You volunteered for community service to help elderly neighbors with chores in their homes. When you enter the house of Mr. Johnson, you find him on the living room floor. There are no signs of bleeding or open wounds and no one else is in the house. Based on your first aid training, take the steps necessary in this situation on the manikin to*  **assess**  *and*  **react upon**  *the situation*.”

This scenario was chosen to sketch a hypothetical scenario that required participants to perform CPR on the manikin. Specifically, it stated that they are alone, making it necessary to act and not rely on additional first aid helpers. Any bleeding and wounds were excluded to ensure that participants do not waste time to check for these. Participants were tested on their performance of the necessary actions required according to the European Resuscitation Council (ERC) guidelines (Soar et al., 2015). For perfect performance, participants had to correctly remember and complete a sequence of actions: check responsiveness, check for breathing and open airways, alert emergency services, and determine correct hand position for compressions. They then alternated between 30 compressions and two rescue breaths. Participants were stopped after administering four cycles of compressions and rescue breaths to avoid fatigue. We refer to this procedure (i.e., initial steps followed by four rounds of 30-2) as one run-through of CPR.

The declarative steps were scored by the experimenter using a pen and paper checklist. The administration of compressions and rescue breaths was recorded on the manikin’s SimPad, a digital tablet device linked with the manikin’s sensors. This procedure — scoring of steps taken followed by one run-through of CPR — was identical for all components marked “CPR Test” in Figure 1 and will be referred to as a *complete* run-through.

##### CPR Video and Quiz I

After the initial assessment, participants were re-trained. First, participants watched a short instructional video specifically made for this research project (see https://osf.io/9er6g/) demonstrating the initial steps, as well as instructions on how to correctly apply chest compressions and rescue breaths. To test whether participants understood and remembered the CPR procedure they completed a multiple-choice quiz about the material presented in the video.^4^

##### S&C Tapping (random)

Subsequently, participants completed six trials of the Synchronization and Continuation (S&C) Tapping Task (based on Semjen et al., 2000). One trial consisted of a synchronization phase and a continuation phase. In the first, 32 recurring short beeps (10ms long) were played with an inter-stimulus interval (ISI) randomly sampled per trail (i.e., random on each trial but fixed for the 32 beeps within a trial) from a uniform distribution between 400 and 800ms. The participants had to tap along with the beep on a trackpad until 32 key presses were recorded (“synchronization” phase). Then the beeps stopped and the participants were instructed to continue tapping another 32 times at the same rate (“continuation” phase). Performance was expressed as the difference between the participant’s interval between the current and previous tap, and the ISI valid for this trial.

##### CPR Compressions I

Following this, participants had the opportunity to practice compressions on the manikin with its live feedback option enabled for one minute. That is, while performing chest compressions, participants could track their depth and frequency on the SimPad and adjust if necessary.

##### S&C Tapping (110bpm) I

Following this, another set of six trials of the S&C tapping task were administered with a fixed ISI of 600ms, which is equivalent to the correct compression rate during CPR (110bpm).

##### CPR Test I.2 and I.3 + FB

Participants were instructed to “*Perform the complete procedure you saw in the video, with four rounds of compressions and rescue breaths*” twice, while their performance was scored as outlined under *CPR Test I.1*. In addition, participants received verbal feedback after each test. If the score of *CPR Test I.3* was below 75%, an additional run-through of CPR was completed.

After the run-throughs of CPR, participants completed questionnaires to gather demographic information, the date their driver’s license was issued, and the approximate number of months between completing their CPR training and obtaining their license. The time between the mandatory training and obtaining the driver’s license ranged from 1 to 60 months (mean = 9.92 and SD = 12.71).

##### Word_I_ Learning

Participants spent 15 minutes studying 35 Swahili-English word pairs. The word pairs were taken from Nelson and Dunlosky (1994) and two unique 35-item sets were created, one each for Session I and II. During word learning, word-pairs were introduced in random order and subsequent repetitions were governed by an adaptive scheduling algorithm (van Rijn, van Maanen, & van Woudenberg, 2009) based on a computational model of human memory that – given previous exposure to a fact – estimates the probability of a correct retrieval during word learning (also see Pavlik & Anderson, 2008). Word-pairs were repeated when the algorithm estimated that answers were almost forgotten, thus balancing the benefits of the spacing and testing effects (for technical details, see Sense et al., 2016). The model’s parameters are continuously adjusted as responses during word learning are collected and compared to the model’s predictions. These item-level model parameters can subsequently be aggregated for each participant and serve as an individual differences measure that we call *rate of forgetting* (Sense et al., 2018). Because the repetitions were adaptively scheduled based on participants’ responses during word learning, some participants will not have seen all word-pairs and the number of repetitions per word-pair varies between participants.

##### Raven Matrices I

Participants performed a shortened version of Raven’s advanced progressive matrices (Raven, 2000). Instead of seeing all 36 matrices without a time limit, participants had 10 minutes to solve half of them (as in Foster et al., 2015). Participants saw the 18 odd-numbered items in the first session (*Raven Matrices I*). Materials were taken from (Loesche, Wiley, & Hasselhorn, 2015).

##### CPR Test I.4

Following the computerized tasks, participants were asked to do another complete run-through of CPR. If the score was below 75%, participants were re-trained (by giving feedback of what needed to improve) and re-tested until they reached criterion (with maximum of two additional iterations, and participants self-paced the start of the re-tests to take breaks if necessary).

##### Word_I_ Recall I

The first session ended with a retention test of the Swahili words. The cued recall test listed all Swahili words that could be studied during *Word_I_ Learning* and participants could respond in any order without a time limit. No feedback was provided.

#### Session II

Participants spent 15 minutes studying a new set of 35 Swahili-English word pairs using the adaptive method described above (*Word_II_ Learning*). Then, participants completed six trials of the S&C tapping task with a fixed ISI of 600ms (*S&C Tapping* (*110bpm*) *II*) followed by the 18 even-numbered items of the shortened version of Raven’s advanced progressive matrices (*Raven Matrices II*). Next, participants were asked to do another complete run-through of CPR (*CPR Test II*) which was repeated if performance was below 75%. Lastly, participants completed the same kind of cued recall test for both the words from Session I (*Word_I_ Recall II*) and Session II (*Word_II_ Recall II*).

#### Session III

The third session started with another complete run-through of CPR (*CPR Test III*), followed by one minute of compressions (*CPR Compressions III*) without live feedback. Then participants were asked to administer rescue breaths until two consecutive ventilations were correctly performed (*CPR Ventilations III*). Next, the CPR multiple choice quiz from Session I was repeated (*CPR Quiz III*). Finally, participants completed the word retention test for the stimulus set of Session I (*Word_I_ Recall II*) and the stimulus set of Session II (*Word_II_ Recall II*).

## Available Data

Here, we will present in more detail the data available for each task, especially for the CPR components, as these are the most complex and novel. Each of the following sections corresponds to a file in the “Individual Tasks” folder in the OSF repository (https://osf.io/m8bxe/) and the labels used to refer to the components are based on Figure 1. The supplement contains more information on the raw data and demonstrates how each component discussed below was computed.

### CPR Performance

All interactions of all participants with the manikin were recorded on the SimPad, resulting in detailed information. Performance scores for chest compressions and rescue breaths were based on Laerdal’s proprietary scoring algorithm (ranging from 0 to 100%; a score of 75% or higher is considered “proficient”). We selected “Guideline type Adult” of the “2010 (EU) – ERC” European Resuscitation Council guidelines in the configuration settings of the SimPad, to ensure that the scoring algorithm was based on criteria set by the recommendations of the ERC. The SimPad outputs overall scores for compressions, ventilations, and a combined score. Those scores as well as a plethora of low-level information is stored in six categories, the first three of which are always the same and the last three vary with the number of recorded events. Table 1 provides an overview and some examples. All participants had previous first aid training. The approximate date of that training and whether they had additional training since was also recorded.

This allows extraction of: High-level performance metrics computed by the Laerdal software (e.g., the *overallScore* used to determine proficiency) and low-level information for each recorded compression and ventilation event, such as the exact depth of individual compressions (*compDepth* in mm) or the volume of each rescue breath (*ventVolume* in ml).

**Figure 2:**
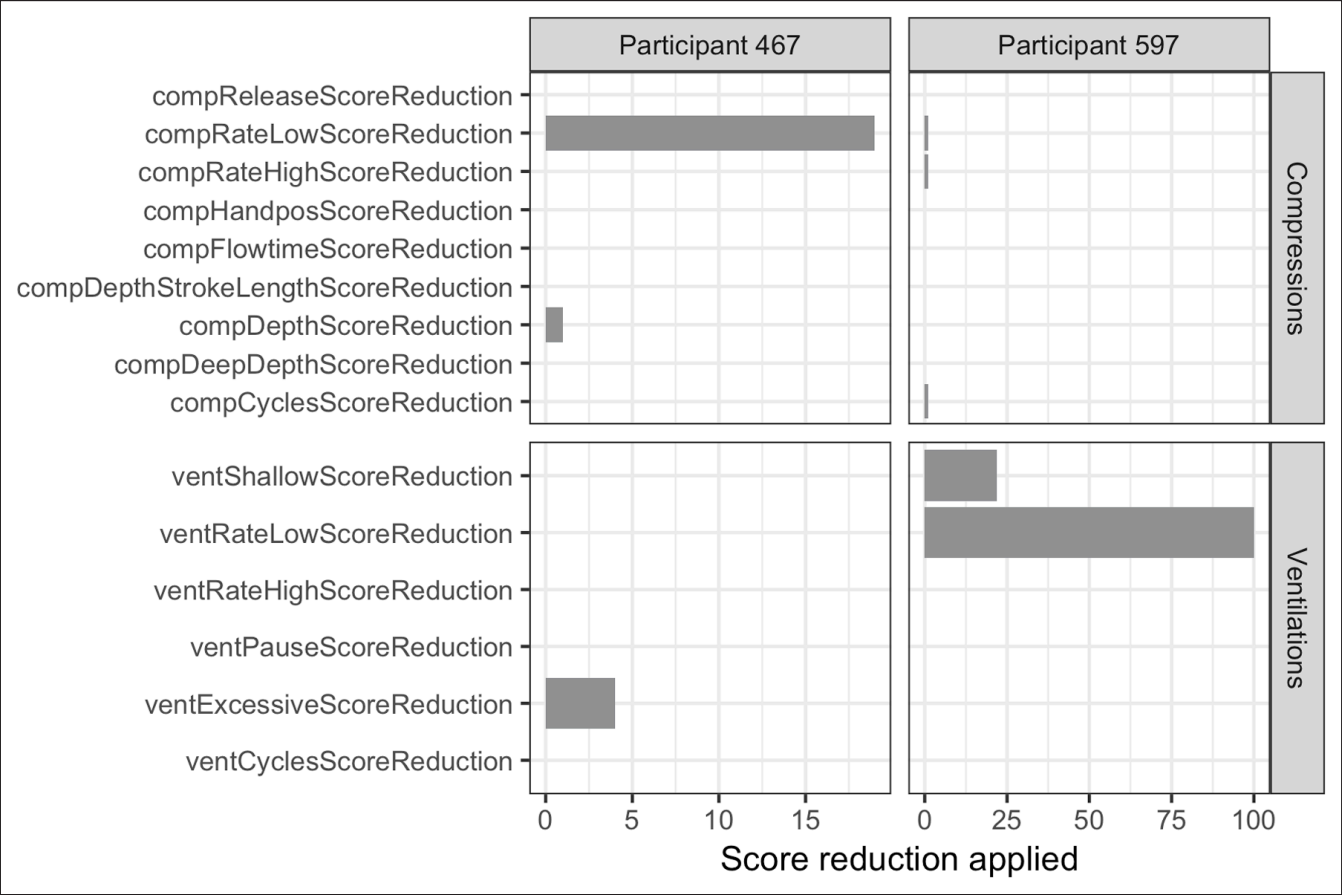
An example of the reductions applied to the scoring for two participants performing a complete run-through of CPR during *CPR TEST I.2+FB*. The labels on the y-axis correspond to entries in the raw data in the column ***type2*** (also see Table 1).

The scoring algorithm implemented on the SimPad starts everyone with a perfect score and subsequently applies reductions based on non-optimal behavior. Figure 2 illustrates the level of detail that is available for two example participants at a single measurement point (*CPR.TEST.I.2+FB*). Participant 467 received score reductions because their compressions were too slow and occasionally not deep enough (overall compression score: 80). Their ventilations were near perfect, with slight score reductions for excessive rescue breath volume (overall ventilation score: 96). The combined overall score is 84 for this participant. The other example participant has a combined score of 73, which is a combination of a near-perfect overall compression score (97) but an overall ventilation score of 0 because the rate with which ventilations were administered was too low (and also did not have enough volume).

Data are available for each individual compression event. Therefore, one could dig even deeper to determine, for example, when exactly Participant 467’s compressions were too slow and by how much: Did they gradually become slower? Or did they consistently compress at a rate that was just at the threshold and sometimes went under?

**Figure 3:**
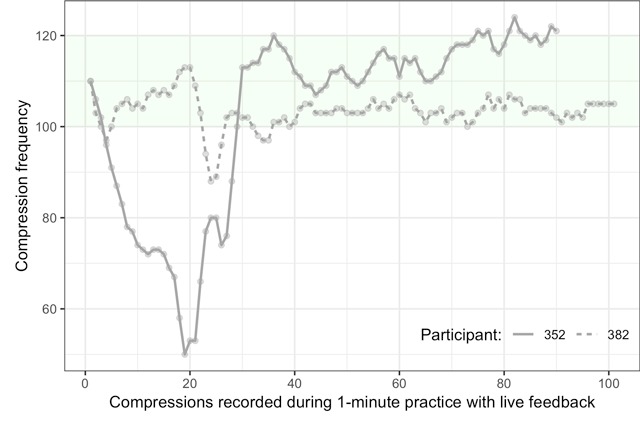
Compression frequencies recorded during CPR Compressions I for the most and least stable participants.

Figure 3 illustrates how such questions could be addressed. Shown is the compression frequency associated with each recorded compression during the 1-minute isolated practice with live feedback in Session I (CPR Compressions I). We isolated the participants exhibiting the most and least stable frequencies, where the stability was quantified as the magnitude of the slope of a linear regression line fit to each participant’s data. Note that this assumes a fairly linear performance profile, which seems to hold in this dataset. More elaborate measures would be needed if, for example, u-shaped performance profiles are expected. As can be seen in the figure, participant 382 shows a very stable pattern with frequencies mostly between 100 and 110, while participant 352’s frequencies decrease over the first 20 compressions only to increase for the next 20. Towards the end, 352 is a bit too fast. The compression scores associated with these two performances are 87 and 99, respectively.

This is but a small example of the extreme richness of the CPR data that are available for all participants at various test moments. The supplement provides a more detailed overview and how to extract relevant aspects of the data.

### Declarative CPR Performance

The Laerdal manikins cannot measure the steps that should be taken to ensure that initiating CPR is appropriate (i.e., checking breathing, alerting emergency services, etc.). The separate scoring (see *CPR Test I.1* in Methods above for details) provides information on whether the existence and ordering of a step was recalled and completed correctly and scores are available for all *CPR Test* components in Figure 1 (i.e., complete run-throughs). We refer to these as *declarative* CPR performance to contrast it with the administration of compressions and rescue breaths. The information thus recorded would allow us to, for example, compute which steps were most likely to be forgotten or how long breathing was checked when it was checked (see supplement).

### Word Learning

Word-pair study time was fixed at 15 minutes for all participants. Word-pairs were introduced in random order and repetition schedules were personalized to each participant using the adaptive algorithm outlined above. For each participant, time-stamped information for each response includes accuracy, latencies for first and confirming keystrokes, and the estimated model parameters.

**Figure 4:**
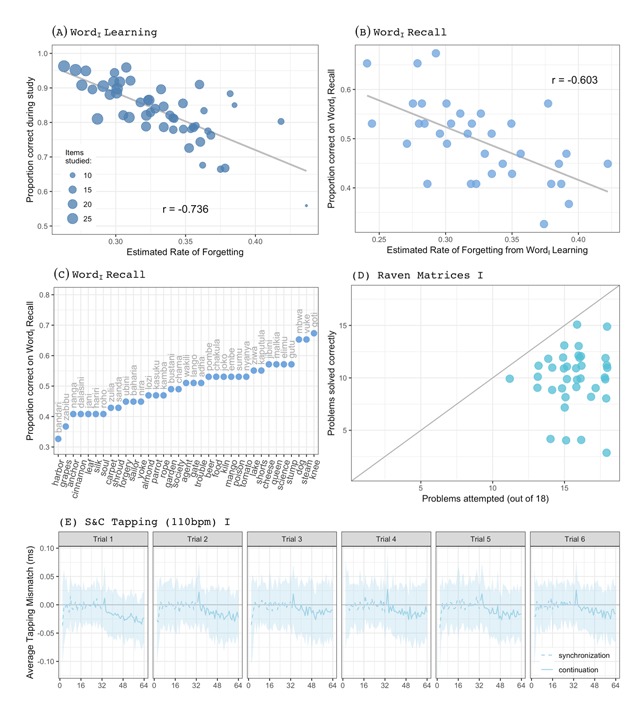
An overview of the type of data that is available for various components. Plot titles refer to labels used in Figure 1. Details are provided in-text. All plots based on data from Session 1 only.

Figure 4(A) depicts, for each participant, the proportion of correct responses they gave during word learning (y-axis) and how those relate to the *rates of forgetting* estimated from the adaptive scheduling system (procedure is identical to Sense et al., 2016). The strong negative correlation indicates that participants that made more errors during word learning are estimated to forget the studied material more quickly, as one would expect.

### Word Recall

For each participant, information is available for each Word Recall measurement regarding the response they gave on each item. Specifically, the file contains information on the Swahili cue, the correct English translation, and the answer provided to each item — and whether the answer was correct. Participants submitted their Word Recall responses as a batch since all cues were on screen at the same time, which means there are no latencies for individual items.

Each point in Figure 4(B) indicates how a participant’s proportion of correct responses on Word_I_ Recall test (x-axis) relates to the *rate of forgetting* estimated during Word_I_ Learning. The high negative correlation suggests that the rate of forgetting does not only capture individual differences during word learning (cf. Figure 4(A)) but also on a subsequent recall test. Additionally, Figure 4(C) shows how the Swahili-English paired associates could be ranked in difficulty based on participants’ performance on Word_I_ Recall.

### Raven matrices

The Raven matrices were presented sequentially: For each problem, we have information about the response latency, the options that were on screen and how they were arranged, which response option was correct, and which was picked by the participant. Given the 10-minute time limit, not everyone attempted all problems. Figure 4(D) shows the relationship between the number of problems attempted and solved by each participant and indicates that many more problems were attempted than solved. This makes sense given the progressively increasing difficulty of the problems.

### S&C Tapping

Responses to each individual event in the task are available in the raw data file. Asynchronies are the differences between the onset of a beep (or when it would have continued) and each recorded tap (i.e., tapping mismatch). Figure 4(E) shows one possible way to aggregate tapping performance by summarizing the tapping mismatch for the 600ms ISI in Session 1 (i.e., S&C Tapping (110bpm) I). Shown is the average tapping mismatch for each of the 64 events on the 6 trials: The SD is indicated by the ribbon, while the mean mismatch is shown by lines. During the synchronization phase (dashed lines), the mismatch is centered around 0 but as the continuation phase (solid lines) progresses, the average mismatch drifts to negative numbers, suggesting that participants sped up.

## Concluding Remarks

In this manuscript, we describe a dataset that we hope will be useful to other researchers. We believe that the type of data presented here are rarely collected: Performance was measured on a number of established lab tasks as well as a complex, real-life task (CPR) across three sessions. Given the richness of these data, we hope other researchers can use them to explore individual differences in performance on a complex, real-life task and traditional lab experiments.

## Data Accessibility Statement

All data and scripts are available at https://osf.io/m8bxe/.

## Footnotes

Note that in the publicly available data, age has been categorized as <20, 20–24, and 25+ in line with privacy regulations.

Note that the lab tasks were administered in Sessions I and II.

https://www.laerdal.com/sg/products/simulation-training/resuscitation-training/resusci-anne-qcpr/.

Responses on the multiple-choice quizzes are not discussed in more detail here but the data are available in the OSF repository (https://osf.io/m8bxe/).
